# Post-infective transverse myelitis following *Streptococcus pneumoniae* meningitis with radiological features of acute disseminated encephalomyelitis: a case report

**DOI:** 10.1186/1752-1947-6-313

**Published:** 2012-09-19

**Authors:** Thomas Williams, John Thorpe

**Affiliations:** 1 Addenbrooke’s Hospital, University of Cambridge School of Clinical Medicine, Box 111, Hills Road, Cambridge, CB2 0SP, UK; 2 Peterborough City Hospital, Edith Cavell Campus, Bretton Gate, Peterborough, PE3 9GZ, UK

**Keywords:** Post-infective demyelination, Transverse myelitis, Acute disseminated encephalomyelitis, Pneumococcal meningitis, *Streptococcus pneumoniae*

## Abstract

**Introduction:**

Post-infectious autoimmune demyelination of the central nervous system is a rare neurological disorder typically associated with exanthematous viral infections. We report an unusual presentation of the condition and a previously undocumented association with *Streptococcus pneumonia* meningitis.

**Case presentation:**

A 50-year-old Caucasian woman presented to our facility with an acute myelopathy three days after discharge following acute *Streptococcus pneumoniae* meningitis. Imaging studies of the spine ruled out an infective focus and no other lesions were seen within the cord. Diffuse, bilateral white matter lesions were seen within the cerebral hemispheres, and our patient was diagnosed as having a post-infective demyelination syndrome that met the diagnostic criteria for an acute transverse myelitis. Our patient clinically and radiologically improved following treatment with steroids.

**Conclusions:**

The novel association of a *Streptococcus pneumoniae* infection with post-infectious autoimmune central nervous system demyelination should alert the reader to the potentially causative role of this common organism, and gives insights into the pathogenesis. The unusual dissociation between the clinical presentation and the location of the radiological lesions should also highlight the potential for the condition to mimic the presentation of others, and stimulates debate on the definitions of acute transverse myelitis and acute disseminated encephalomyelitis, and their potential overlap.

## Introduction

Myelopathy is a relatively common neurological presentation characterized by the triad of motor, sensory and autonomic features bilaterally affecting the lower limbs and extending a variable distance up the thorax, typically producing a rostral sensory level. Acute transverse myelitis (ATM) is a rare cause of myelopathy, with an incidence of one to four cases per million per year [1]. In order to improve accuracy of reporting, research and diagnosis, the Transverse Myelitis Consortium Working Group have produced criteria for the diagnosis of ATM (Table 1). All of the inclusion criteria must be present, and all of the exclusion criteria absent, for a diagnosis of acute transverse myelitis to be made. Once diagnosed, ATM is then classified as either idiopathic (in the absence of all disease associated features), or disease associated (if they are present). There are two key features of the criteria in relation to our patient’s case: firstly, they propose that ATM can be diagnosed in the absence of radiological evidence of spinal lesions if cerebral spinal fluid (CSF) analysis reveals signs of inflammation; and secondly that magnetic resonance imaging (MRI) scan findings of multifocal inflammation within the brain in the presence of ATM results in the classification of disease-associated ATM, as they are thought likely to represent manifestations of either multiple sclerosis (MS), a clinically isolated syndrome (CIS) or acute disseminated encephalomyelitis (ADEM).

**Table 1:** Diagnostic criteria for acute transverse myelitis

**Inclusion criteria**	**Exclusion criteria**	**Disease associated features**
Development of sensory, motor or autonomic dysfunction attributable to the spinal cord	History of spinal irradiation in the last 10 years	Serological or clinical evidence of a systemic autoimmune disorder
Bilateral signs and/or symptoms (not necessarily symmetrical)	Clear arterial distribution of the clinical deficit suggesting anterior spinal artery thrombosis	Central nervous system (CNS) manifestation of syphilis, Lyme disease, HIV, human T-lymphotropic virus 1 (HTLV-1), mycoplasma, other viral infections
Exclusion of extra-axial compressive etiology by magnetic resonance imaging (MRI) or myelography	Abnormal flow void on the surface of the spinal cord consistent with arteriovenous malformation	Abnormalities on brain MRI scans suggestive of multiple sclerosis (MS) or acute disseminated encephalomyelitis (ADEM)
Inflammation within the spinal cord demonstrated by cerebrospinal fluid (CSF) pleocytosisor elevated IgG index or gadolinium enhancement		History of clinically apparent optic neuritis
Peak severity reached between four hours and 21 days after onset		

Table modified from the Transverse Myelitis Consortium Working Group [1]. All of the inclusion criteria must be present, and all of the exclusion criteria absent, for a diagnosis of acute transverse myelitis to be made. In the absence of disease associated features the transverse myelitis is deemed idiopathic; in their presence it is deemed disease associated.

ADEM is a predominantly paediatric disease, where it has an incidence of 0.4 to 0.8 per 100,000. It is even more rare in adults, where it has a mean age of onset of 32 to 51 years [2]. It typically presents with focal neurological signs and encephalopathy; due to the disseminated and variable location of involvement within the central nervous system (CNS), such focal lesions can include motor, sensory, cranial nerve, brainstem or cerebellar signs. Its mechanism is autoimmune, resulting in multifocal demyelination of the CNS, with relative preservation of axons. These foci are mainly in supratentorial white matter, and are pathologically characterised by their perivenous distribution [3]. The thalamus, basal ganglia, cerebellum and spinal cord can also be involved [4]. Differentiating ADEM from a clinically isolated syndrome of multiple sclerosis is often challenging. In contrast to multiple sclerosis, however, the lesions seen in ADEM on MRI are not sharply delineated, and are often very large or confluent [5]. The temporal association with infections and, less commonly, vaccinations, insect stings and immunoglobulin administration has led to our current theories of pathogenesis: antigenic mimicry between pathogenic antigens and myelin epitopes; activation of clonal populations of autoreactive T-cells; suppression of CD4+ T-cells; and stimulation of major histocompatibility II molecule expression in astrocytes and microglia have all been suggested, and it is unlikely that such mechanisms would be mutually exclusive [5].

In order to improve consistency and accuracy in the diagnosis of and research into ADEM, definitions were produced by the International Pediatric MS Study Group [6]. It was emphasised that the diagnosis of ADEM must rest upon the clinical features of an acute or subacute onset of multifocal features representing involvement of different areas of the CNS, together with encephalopathy (defined as behavioural change or alteration in consciousness). The definition also requires MRI features, typically large multifocal or unifocal lesions within the white matter of the brain and/or spinal cord, as described above. Importantly, however, it is suggested that the diagnosis of ADEM cannot be made based upon radiological features alone.

Although the above definition was intended for use only within the paediatric group (age <10 years), it has been applied to adult cases of ADEM. Young *et al*. demonstrated that in a cohort of 13 patients (mean age 43 years) with ADEM diagnoses based upon the pathological hallmark of perivascular demyelination demonstrated on biopsy and/or autopsy, encephalopathy or reduced level of consciousness was seen significantly more often compared to a larger cohort (n=91) showing confluent demyelination (the hallmark of MS *P* <0.001) [7]. Although a small study, this appears to lend support to the International Pediatric MS Study Group’s emphasis upon clinical presentation, and suggests that it may also be applied to adults.

## Case presentation

A 50-year-old, previously well Caucasian woman with a one-week history of flu-like symptoms and three days of purulent exudate from both ears presented to our facility with vomiting, collapse and reduced conscious level. Investigations confirmed a diagnosis of acute *Streptococcus pneumoniae* meningitis secondary to otitis media. Our patient was treated on an intensive care ward, requiring sedation and intubation. Initial treatment was with intravenous amoxicillin and acyclovir, later switching to ceftriaxone. Our patient made a slow but uneventful recovery, leaving intensive care after six days, and being discharged a further five days later.

Our patient reported gradually increasing lower limb weakness at home, and on the third day post-discharge she was readmitted following a fall. On admission she was fully conscious but paraparetic, with impaired sensation in her lower limbs and painless urinary retention. On examination she was apyrexial, with normal fundi, cranial nerves and upper limbs. Examination of the lower limbs revealed slightly reduced tone, a pyramidal pattern of weakness, moderately brisk reflexes and bilateral extensor plantars. A sensory level was present at T10.

Initial investigations revealed a mild neutrophilic leukocytosis. Other routine blood test results were normal.

**Figure 1:**
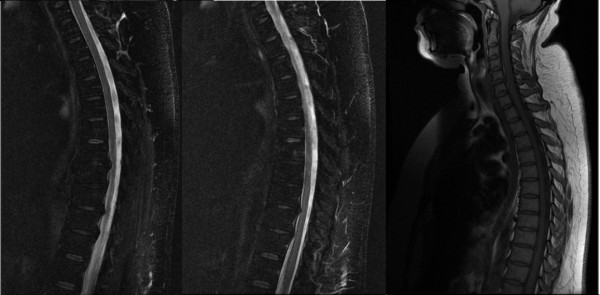
**Magnetic resonance imaging scans of the spinal cord acquired the day after readmission.** Fast short tau inversion recovery (T2-weighted and T1-weighted, (**A**) and (**B**), respectively) and T1-weighted fast spin echo (**C**) sagittal images of the spinal cord are shown: minor degenerative disease can be observed but their is no cord compression from disc or abscess and no obvious intrinsic signal change.

**Figure 2:**
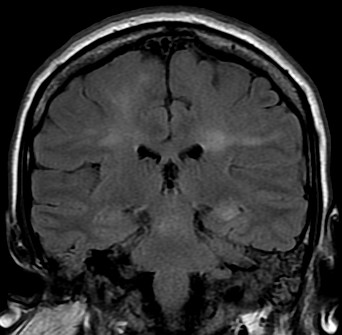
**Magnetic resonance imaging scans of the brain acquired the day after readmission.** The coronal fluid attenuation inversion recovery image shows several ill-defined foci of high T2 signal in the corona radiata of both hemispheres.

An urgent MRI scan of the spinal cord was undertaken to rule out an epidural abscess (Figure 1A-C). No epidural collection or discitis were seen and there was no abnormal signal within the cord. Magnetic resonance venography of the cerebral veins was normal but MRI of the brain revealed several ill-defined foci of high T2 signal in the corona radiata of both hemispheres (Figure 2). Grey-white matter differentiation was retained, and the cortical appearance was normal.

CSF analysis revealed a lymphocytic pleocytosis (12 polymorphs, 96 lymphocytes and 22 red blood cells per mm^3^), a raised protein level (1.84g/L, normal <0.4g/L) and normal glucose level. No oligoclonal bands or organisms were found, virology screen results were negative, and no organisms were cultured.

Having excluded a septic focus and sinus thrombosis, on the basis of the MRI scan appearance, a diagnosis of post-infective demyelination that met the diagnostic criteria of an acute transverse myelitis was made. Our patient received a three-day course of methylprednisolone (1g/day intravenously), followed by oral prednisolone (60mg/day). Repeated imaging of the brain 10 days after readmission showed that the lesions were beginning to resolve. Her power began to improve and she was discharged four weeks after her readmission. Significant recovery was achieved by four months, but with residual deficits: walking was limited to 500 metres unaided, with a spastic gait and mild pyramidal weakness and hyper-reflexia of the lower limbs. There were no further neurological events over the ensuing year by which time she was able to walk two miles but had evidence of a mild residual spastic paraparesis.

## Discussion

We report an unusual case of post-infectious demyelination. The diagnosis was initially slightly tentative based on the presentation of an acute spinal cord syndrome associated with suggestive brain lesions on MRI scan despite a causative cord lesion not being visualised. Initially it seemed more likely that our patient had a pyogenic abscess or conceivably venous sinus thrombosis but these were excluded by appropriate imaging. The response to steroids provided further support for the diagnosis.

Our patient met the Transverse Myelitis Consortium Working Group’s criteria [1] for ATM due to the appropriate clinical presentation, evidence of inflammation within the spinal cord obtained on CSF analysis, and exclusion of other aetiologies; it is worth highlighting that the criteria state that evidence of inflammation on CSF analysis alone, in the absence of radiological features, is sufficient to support the diagnosis. Due to the abnormal brain imaging, however, it could be classified as a disease-associated ATM, with MS, a CIS or ADEM suspected as the underlying pathological process. There were no features of a systemic connective tissue disease.

Post-infectious ATM is a well-recognised phenomenon. It can also occur following vaccinations, and in both cases it is thought that the mechanism involves a breakdown of self-tolerance leading to an autoimmune reaction against CNS antigens [8]. It has been suggested that post-infective ATM is a localised form of ADEM [9,10], and it has also been emphasised that disseminated encephalomyelitis can produce a broad spectrum of pathological and anatomical manifestations [11]. One would expect, however, such cases to be accompanied by evidence of spinal lesions on MRI scans, and such post-infectious or post-vaccination demyelinating disorders of the CNS are usually characterised by multifocal signs and encephalopathy, leading to the diagnosis of ADEM [12].

As previously discussed, despite the presence of large and confluent lesions without sharp delineation on brain imaging being highly suggestive of a diagnosis of ADEM, such a diagnosis cannot be made based upon radiological criteria alone, and hence it is excluded due to the absence of the clinical features. Our patient did not have a history suggestive of previous demyelination, there was an absence of oligoclonal bands and the imaging would not be typical for a clinically isolated syndrome. It also seems highly unlikely that the temporal association with the meningeal infection was a coincidence.

We were therefore left with our patient meeting the criteria for a disease-associated ATM following pneumococcal meningitis, with normal spinal imaging and brain lesions characteristic of ADEM, but without the clinical features necessary for its diagnosis.

## Conclusions

It appears that this case is unusual for a number of reasons. Firstly, to the best of our knowledge, this is the first reported case in the English literature of an *S. pneumoniae* infection being associated with post-infective autoimmune CNS demyelination. In such conditions, the causal infections are most commonly viral, particularly those causing exanthematous disease (for example, measles, rubella or varicella) [2]. Streptococcal infections have previously been associated with ADEM, though this is limited to the Lancefield group A β-haemolytic species. Such paediatric cases are associated with anti-basal ganglia antibodies and a high incidence of basal ganglial lesions, raising similarities with Sydenham’s chorea and the paediatric autoimmune neuropsychological disorders associated with *Streptococcus* (PANDAS) [13]. One adult case of post-group A β-haemolytic streptococcal ADEM has been reported, in which there were no basal ganglia lesions [14].

In contrast to the β-haemolytic species, there have only been two previous Japanese reports of suspected ADEM following α-haemolytic *S. pneumoniae* infection [15,16]. There has also been one report of widespread white matter lesions in association with a paediatric case of *S. pneumoniae* meningitis [17]. In contrast to our patient’s case, however, these occurred very early in the course of the disease, and it was thought that they were likely to represent global ischaemia and cytotoxic oedema secondary to a cerebral vasculitis initiated directly in response to the presence of the pneumococcal antigens. This is in contrast to the proposed mechanisms thought to be causative in the post-infective demyelination of our case, which typically occurs after a delay of a few days to weeks from the initiating infection as a result of direct autoimmune attack of CNS antigens.

The involvement of *S. pneumoniae* in the initiation of post-infective demyelination has implications for the pathogenesis. Proposed pathogenic mechanisms by which streptococcal infections cause post-infective demyelination include involvement of the mentioned anti-basal ganglia antibodies [13], and stimulating clonal expansion of anti-myelin T cells via exotoxin superantigens produced by group A β-haemolytic strains [18]. This case therefore highlights not only how pneumococcal infections should be considered in the aetiology of post-infective demyelination, but also, as neither the anti-basal ganglia antibodies nor the superantigenic exotoxins are associated with pneumococcal infections, how a variety of pathological mechanisms must be capable of leading to post-streptococcal demyelination.

The second unusual feature of this case is the apparent overlap between the clinical features of ATM and the radiological features of cerebral ADEM. The precise nature of the lesions is unlikely to be determined; if histological specimens were available, the finding of a perivascular distribution of the demyelination would give a pathological diagnosis of ADEM, lending support to the radiological appearance and suggesting that the absence of encephalopathy is not significant. This could then be classified as ADEM presenting as a disease-associated ATM. Indeed, in a previous study of patients with histologically confirmed ADEM, encephalopathy and depressed level of consciousness were only 77% and 62% sensitive, respectively [7]. In the original consensus definition of ADEM, the International Pediatric MS Study Group also acknowledged that the absolute requirement for encephalopathy may be overly restrictive, but deemed it necessary in order for sufficient specificity [6].

If this were to be the case, the disparity between clinical and radiological localisation of the lesions would still be unusual for ADEM. Despite our patient’s signs and symptoms pointing to a clinical diagnosis of acute transverse myelitis with a T10 sensory level, imaging revealed lesions limited to the corona radiata. Awareness of this will lead to improved diagnosis, as it highlights how the condition may mimic the presentation of others, and the importance of imaging the whole CNS once the more common differentials have been ruled out.

## Consent

Written informed consent was obtained from the patient for publication of this case report and any accompanying images. A copy of the written consent is available for review by the Editor-in-Chief of this journal.

## Competing interests

The authors declare that they have no competing interests.

## Authors’ contributions

TW compiled the case report and conducted the literature review. JT was responsible for the care of the patient and provided guidance and advice throughout the writing of the report. Both authors read and approved the final manuscript.

## References

[B1] Transverse Myelitis Consortium Working Group Proposed diagnostic criteria and nosology of acute transverse myelitis Neurology 2002 59 499 505 12236201 10.1212/wnl.59.4.499

[B2] Sonneville R Klein I de Broucker T Wolff M Post-infectious encephalitis in adults: diagnosis and management J Infect 2009 58 321 328 10.1016/j.jinf.2009.02.011 19368974 PMC7125543

[B3] Lassmann H Acute disseminated encephalomyelitis and multiple sclerosis Brain 2010 133 317 319 10.1093/brain/awp342 20129937

[B4] Wender M Acute disseminated encephalomyelitis (ADEM) J Neuroimmunol 2011 231 92 99 10.1016/j.jneuroim.2010.09.019 21237518

[B5] Tselis A Lisak R Acute disseminated encephalomyelitis Clinical Neuroimmunology 2005 2 Oxford University Press, Oxford, UK 147 171

[B6] Krupp LB Banwell B Tenembaum S International Pediatric MS Study Group Consensus definitions proposed for pediatric multiple sclerosis and related disorders Neurology 2007 68 S7 S12 10.1212/01.wnl.0000259422.44235.a8 17438241

[B7] Young NP Weinshenker BG Parisi JE Scheithauer B Giannini C Roemer SF Thomsen KM Mandrekar JN Erickson BJ Lucchinetti CF Perivenous demyelination: association with clinically defined acute disseminated encephalomyelitis and comparison with pathologically confirmed multiple sclerosis Brain 2010 133 333 348 10.1093/brain/awp321 20129932 PMC2822631

[B8] Agmon-Levin N Kivity S Szyper-Kravitz M Shoenfeld Y Transverse myelitis and vaccines: a multi-analysis Lupus 2009 18 1198 1204 10.1177/0961203309345730 19880568

[B9] AlDeeb SM Yaqub BA Bruyn GW Biary NM Acute transverse myelitis - a localized form of postinfectious encephalomyelitis Brain 1997 120 1115 1122 10.1093/brain/120.7.1115 9236624

[B10] Ginsberg L Disorders of the spinal cord and roots Pract Neurol 2011 11 259 267 10.1136/practneurol-2011-000069 21746716

[B11] Brinar VV Poser CM The spectrum of disseminated encephalomyelitis Clin Neurol Neurosurg 2006 108 295 310 10.1016/j.clineuro.2005.11.017 16387421

[B12] Brinar VV Habek M Zadro I Barun B Ozretic D Vranjes D Current concepts in the diagnosis of transverse myelopathies Clin Neurol Neurosurg 2008 110 919 927 10.1016/j.clineuro.2008.07.002 18718707

[B13] Dale RC Church AJ Cardoso F Goddard E Cox TC Chong WK Williams A Klein NJ Neville BG Thompson EJ Giovannoni G Poststreptococcal acute disseminated encephalomyelitis with basal ganglia involvement and auto-reactive antibasal ganglia antibodies Ann Neurol 2001 50 588 595 10.1002/ana.1250 11706964

[B14] Ning MM Smirnakis S Furie KL Sheen VL Adult acute disseminated encephalomyelitis associated with poststreptococcal infection J Clin Neurosci 2005 12 298 300 10.1016/j.jocn.2004.03.030 15851086

[B15] Ueda M Kanamori A Mihara T Hara H Mutoh T A case of acute disseminated encephalomyelitis (ADEM) following treatment for pneumococcal meningoencephalitis [in Japanese] Rinsho Shinkeigaku 2009 49 96 99 10.5692/clinicalneurol.49.96 19348173

[B16] Ohnishi H Sawayama Y Ariyama I Yamaji K Furusyo N Hayashi J Acute disseminated encephalomyelitis (ADEM) onset during meningitis and sepsis [in Japanese] Kansenshogaku Zasshi 2007 81 577 581 17966640 10.11150/kansenshogakuzasshi1970.81.577

[B17] Jorens PG Parizel PM Wojciechowski M Laridon A De Weerdt A Mertens G Ceulemans B Streptococcus pneumoniae meningoencephalitis with unusual and widespread white matter lesions Eur J Paediatr Neurol 2008 12 127 132 10.1016/j.ejpn.2007.06.007 17881267

[B18] Jorens PG VanderBorght A Ceulemans B Van Bever HP Bossaert LL Ieven M Goossens H Parizel PM Van Dijk H Raus J Stinissen P Encephalomyelitis-associated antimyelin autoreactivity induced by streptococcal exotoxins Neurology 2000 54 1433 1441 10.1212/WNL.54.7.1433 10751252

